# Long COVID: Lived Experiences, Diagnosis, Treatment and Care

**DOI:** 10.1111/hex.70757

**Published:** 2026-07-05

**Authors:** Jordan Mullard

**Affiliations:** ^1^ Population Health Sciences Institute Newcastle University Newcastle‐Upon‐Tyne UK

1

When we began the journey for our Special Issue into Long COVID, we could not have anticipated the volume of response we've received. We have had over 100 submissions and have accepted and published 42. The breadth and scope of this research encapsulate the range of experiences, voices and perspectives on Long COVID from a variety of contexts. Our Special Issue provides an authoritative account of the latest knowledge on Long COVID. It does this from a similarly broad spectrum of academic disciplines ranging from the social sciences (and the use of ethnographic approaches) to epidemiological and biomedical studies. What is more, we hear from a range of perspectives including people living with Long COVID to health workers and practitioners supporting patients, through to people leading policy initiatives and defining the field of diagnosis and treatment. Further still the special issue includes research from a global perspective with studies from Brazil, Canada, Australia, Europe, The Americas and China. Whilst I can't do justice to all 42 papers in this editorial, I have selected 15 that showcase some of this phenomenal breadth.

Strong themes that emerge across the Special Issue include the lived experience of Long COVID, the psychological and social consequences of persistent symptoms, the role of peer and community support, inequalities in access to care and the development of new interventions and service responses. Taken together, these studies demonstrate that Long COVID is not only a biomedical condition but also a social, psychological and healthcare challenge that continues to reshape lives, services and systems.

Several studies foreground the experiences of people living with Long COVID and the profound disruption the condition can have on everyday life. Across diverse settings, participants described persistent and fluctuating symptoms, uncertainty about recovery, disruption to employment and social participation, and challenges maintaining previous identities and roles [1, 2, 3, 4]. These experiences frequently extended beyond physical symptoms to encompass emotional distress, loss, frustration and concerns about the future [5, 6]. The psychological burden associated with Long COVID emerged as a particularly important theme, with studies highlighting anxiety, uncertainty and the cumulative impact of living with an often poorly understood condition [5, 6].

Alongside these challenges, the studies reveal the importance of support networks in helping people navigate illness and recovery. Informal support from family, friends and communities was frequently described as essential, particularly when formal healthcare services were difficult to access or unable to meet people's needs [7]. Peer‐support initiatives and digital communities emerged as important spaces for validation, information exchange and collective sense‐making, enabling individuals to share experiences and develop practical strategies for coping with persistent symptoms [8, 9]. These findings highlight the important role of experiential knowledge in shaping understandings of Long COVID and supporting those living with the condition.

A further theme concerns the relationships between people living with Long COVID and healthcare systems. Several studies describe difficulties obtaining recognition, diagnosis and appropriate support, with participants reporting experiences of uncertainty, dismissal and fragmented care pathways [2, 3, 8, 10]. Trust emerged as a critical component of these interactions, shaping experiences of healthcare engagement and influencing how individuals navigated transitions between services and sources of support [11]. Consumer‐focused research similarly highlighted gaps between patient expectations and service provision, reinforcing calls for more responsive and person‐centred models of care [4].

Importantly, the evidence also demonstrates that experiences of Long COVID are not distributed equally. Studies exploring intersectionality and inclusion illustrate how experiences are shaped by overlapping social, economic and demographic factors, with some groups facing additional barriers to diagnosis, care and participation [12, 13]. These findings are echoed by epidemiological analyses demonstrating inequalities in recorded diagnosis and access to timely care, raising important questions about unmet need and the visibility of Long COVID within healthcare systems [10]. Together, these studies emphasise the need for equitable and inclusive approaches to both research and service provision.

Encouragingly, several contributions move beyond documenting challenges to explore potential solutions. Co‐produced interventions, rehabilitation programmes and innovative support models demonstrate the value of involving people with lived experience in the design and delivery of services [14, 15]. Such approaches recognise the complexity of Long COVID and seek to address not only physical symptoms but also the psychological and social dimensions of recovery. Across the Special Issue, there is a clear consensus that future services must be multidisciplinary, inclusive and responsive to the diverse experiences of those living with Long COVID.

The international breadth of this collection is particularly noteworthy. Drawing on research conducted across the United Kingdom, Brazil, North America, Australia and other international contexts, the studies reveal both commonalities and important contextual differences in experiences of Long COVID. Despite variations in healthcare systems, policy environments and service provision, recurring themes of uncertainty, recognition, support, trust and inclusion are evident across settings. This consistency suggests that many of the challenges faced by people living with Long COVID transcend national boundaries, while also highlighting the value of international learning in developing effective and equitable responses to this emerging condition.

Perhaps one of the most striking features of the Long COVID field is the central role played by people living with the condition in shaping its development. Long before formal diagnostic criteria, clinical pathways and treatment programmes emerged, individuals experiencing persistent symptoms documented their experiences, formed communities, generated collective knowledge and advocated for recognition [2]. The studies featured within this Special Issue reflect the continuing importance of lived experience in shaping research, services and policy responses. Together, they demonstrate that understanding Long COVID requires not only biomedical investigation but also sustained attention to the voices, experiences and expertise of those living with its consequences.

## Funding

The author has nothing to report. However, Mullard's research into Long Covid was part of the NIHR LOCOMOTION project based at Leeds Univeristy.

## Conflicts of Interest

The author declares no conflicts of interest.

## Data Availability

The data that support the findings of this study are available from the corresponding author upon reasonable request.
