# Correction to “Long COVID: Lived Experiences, Diagnosis, Treatment and Care”

**DOI:** 10.1111/hex.70778

**Published:** 2026-07-27

J. Mullard, “Long COVID: Lived Experiences, Diagnosis, Treatment and Care,” *Health Expectations* 29 (2026): e70757. https://doi.org/10.1111/hex.70757.

The affiliation of the author Jordan Mullard was incorrect and has been corrected to: Centre for Philosophy of Epidemiology, Medicine and Public Health, University of Johannesburg, UK

We apologize for this error.

